# Genetics of bilateral pediatric cataract in the Israeli and Palestinian populations

**DOI:** 10.1007/s00417-024-06546-2

**Published:** 2024-06-14

**Authors:** Claudia Yahalom, Irene Anteby, Karen Hendler, Tamar Harel, Avital Eilat, Michal Macarov

**Affiliations:** 1 Faculty of Medicine, Hebrew University of Jerusalem, Jerusalem, Israel; 2 Department of Ophthalmology, Hadassah Medical Center, Jerusalem, 12000 POB Israel; 3 Department of Genetics and Metabolic Diseases, Hadassah Medical Center, Jerusalem, Israel

**Keywords:** Pediatric cataract, Congenital cataract, Inherited cataract, Genetics of cataract

## Abstract

**Purpose:**

Bilateral pediatric cataract (BPC) is one of the most common causes of childhood visual impairment and blindness worldwide. A significant percentage of pediatric cataracts are caused by genetic alterations. We aim to characterize the set of genes and variants that cause BPC in the Israeli and Palestinian populations and to assess genotype-phenotype correlation.

**Methods:**

Retrospective study in a multidisciplinary center for visual impairment, located in a tertiary medical center. Medical charts of families who underwent genetic counseling because of BPC in a family member were reviewed. Clinical characteristics and genetic tests results were obtained from medical records of affected subjects.

**Results:**

Twenty-two families (47 patients) underwent genetic counseling and completed genetic testing. Causative variants were identified in 18/22 (81.8%) of the families, including 3 novel variants. Genetic testing used included mainly panel for congenital cataracts and whole exome sequencing. Eleven families performed genetic testing with the intention of future pregnancy planning. Main causative genes identified were crystalline genes followed by transcription factor genes. *BCOR* gene variants were associated with persistent fetal vasculature (PFV) cataract in two of three families.

**Conclusions:**

Combined molecular techniques are useful in identifying variants causing pediatric cataracts and showed a high detection rate in our population. *BCOR* gene variants might be associated with PFV type of cataracts. The study of pathogenic variants may aid in family planning and prevention of pediatric cataracts in future pregnancies. Additionally, in certain cases, it assists in diagnosing non-suspected syndromic types of pediatric cataracts.

**Supplementary Information:**

The online version contains supplementary material available at 10.1007/s00417-024-06546-2.

## Key message

***What is known***

• Bilateral pediatric cataracts are often inherited.

• Detection rate of causative variants is high.

• Correlation between genes and phenotype is still scarce.

***What is new***

• First evidence of possible correlation between *BCOR* gene and persistent fetal vasculature cataract.

• Additional data supporting recently published relationship between *GJA3* gene variant (c.199G > T) and glaucoma.

• Importance of genetic testing in diagnosing “non-suspected syndromic types of pediatric cataracts”.

• Three novel causative variants identified.

**Supplementary Information:**

The online version contains supplementary material available at 10.1007/s00417-024-06546-2.

## Introduction

Bilateral pediatric cataract (BPC) is a common cause for childhood blindness accounting for 10-to-20% of reported cases worldwide [1]. Although BPC is a relatively rare condition, it can cause severe visual impairment in around 40% of cases even when treated timely with modern surgical techniques [2]. 

Based on a systematic review the prevalence of cataracts in childhood was estimated to range from 1.91 to 4.24 per 10,000, with the highest numbers in Asia reaching 7.43 per 10,000 [3]. In the United kingdom prevalence was reported as 3.18 per 10,000 by 5 years of age [4]. 

BPC may occur as an isolated pathology, may be associated with other ocular developmental abnormalities, or as part of a systemic condition [3, 5, 6]. Approximately 25-50% of the pediatric cataracts are inherited [3, 7]. The majority of the inherited cataracts are autosomal dominant while autosomal recessive and X-linked inheritance patterns have also been reported [8, 9]. Up-to date approximately 100 genes and around 200 loci have been identified as causatives of BPC (“Cat-Map” website (http://cat-map.wustl.edu/) [5, 9], suggesting the lack of a main gene accounting for a high percentage of cases [8]. 

Attempts have been made to find a phenotype that points to a specific affected gene, but similar cataract morphologies can be associated with different gene variants and different cataract types can be identified in a given gene variant, thus limiting efforts to make informative genotype-phenotype correlations [5]. 

The detection rate for genetic variants has been reported to be around 75%, based on gene panels using next generation sequencing (NGS) [10]. 

The aim of our study was to report the variety of gene variants (novel and recurrent) causing BPC and the detection rate for these variants in our population. We also aimed to look for any correlations between cataract characteristics and the causing gene variants.

## Methods

Data were collected from medical charts of patients seen in a low vision clinic located at a tertiary University Hospital. This multidisciplinary clinic includes pediatric ophthalmologists, low vision optometrists, a social worker and genetic counselors specialized in inherited eye diseases (IED). Patients with visual impairment from all over the country are referred to our clinic in order to benefit from the multidisciplinary approach and since, in most of them, an IED is the cause for the impaired vision, the family may undergo genetic counseling and testing if interested.

Data were taken from medical charts of patients who underwent genetic counseling from January 2015 to July 2023 and included children with impaired vision due to cataract or adults who came for genetic testing due to BPC in the family.

Details extracted from medical charts included demographic data, cataract characteristics, associated ocular and systemic conditions as well as genetic tests results. Information regarding the genetic analyses performed including molecular tests used, was also retrieved from medical files. The pathogenicity of each variant was determined by the lab [Invitae or Hadassah genetic lab] according to ACMG 2015 guidelines [11, 12]. Only pathogenic or likely pathogenic gene variants were considered in our study.

## Results

Our study included 47 patients (27 female and 20 male) from 22 unrelated families. Eighteen families were of Jewish origin (12/18 were Ashkenazi, followed by incidence by Morocco and Kurdistan Jewish, Table 1) and four were of Arab Muslim origin. Age at genetic testing ranged from 3 months to 43 years.

**Table 1:** Clinical and molecular data of patients with bilateral pediatric cataract who underwent genetic testing

Family/ Patient Number	Ethnicity	Inheritance	Family history	Test done	Gene	Nucleotide change	Predicted amino acid change	Glaucoma	Cataract description	Segregation	Variant classification	Gender/age at genetic testing in index patient	Novel/reported variant
FAM1 -I:1 -II:1	Ashkenazi	AD	-	Sanger	*PAX6*	c.38G > T	p.G13V.	-No -No	-N/A -anterior dot capsular cataract. No surgery.	Maternal: (mother mosaic)	Pathogenic	-Female -Male/1 year	Martha et al. [13]
FAM2 -I:1 -II:1	Ashkenazi	AD	+	Exome	*MIP*	c.80G > A	p.G27E	-No -No	-N/A -Dense nuclear (Surgery: age 1 year) *	De novo	Likely pathogenic	-Female -Female/ 32 years	Novel
FAM3 -I:1 -II:2	Kurdistan-Morocco Jewish	X-linked Dominant	+	CMA (validation by MLPA)	*BCOR*	20kbp Deletion in the BCOR gene Xp11.4(39,918,361 − 39,937,208)X1mat		-Yes -Yes	PFV-microphthalmia ASD -sx age 2 months	Maternal (mosaic)		-Female -Female -Female /1 year	Hu et al. [14]
FAM4 -I:1 -II:1	Ashkenazi	AD	+	Exome	*CRYGD*	c.401_402dup	p. Y134fs	-No -No	-N/A -N/A sx age 8 months *	Maternal (affected)	Pathogenic	-Female -Female/34 years	Santhiya et [15]
FAM5 -I:1 -II:1	Jewish	X-linked Dominant	+	Exome	*BCOR*	c.3490 C > T	p.(R1164*)	-Yes -Yes	-N/A *	Maternal (affected)	Pathogenic	-Female -Female/age 32 years	Hilton et al. [16]
FAM6 -I:1 -II:2	Ashkenazi	AD	+	Panel	*CRYBA1*	c.215 + 1G > A (Splice donor),		N/A	-N/A *	Paternal (affected)	Pathogenic	-Male -Male -Male/1 year	Kannabiran et al. [17]
FAM7 -I:1 -II:1	Muslim	AD	+	Panel	*MIP*	c.606G > A	p.(W202*)	N/A	-N/A *	Paternal (affected)	Pathogenic	-Male/ 31 years -Male	Reis et al. [18]
FAM8 -II:1	Tunisian Jewish	Unknown	+	Panel	No gene			-No	Cataract, retinal detachment *	No gene identified		-Male/53 years	
FAM9 -II:1	Ashkenazi	AD	-	Panel	*CRYBB2*	c.61_63del	p.I21del	N/A	-N/A. Surgery first year of life	N/A	Likely pathogenic	Male/1 year	Novel
FAM10 -II:1	Kurdistan Jewish	Unknown	+	Exome	No gene			N/A	-N/A. *	No gene identified		-Female/35 years	
FAM11 -II:1	Ashkenazi	AD	+	Panel	*CRYBA1*	c.272_274del	p.G91del	-Yes	-N/A -Dense nuclear- Sx age 2 months	N/A	Pathogenic	-Female/12 years	Mohebi et al. [19]
FAM12 -II:2	Muslim	AR	+	Exome	*CRYBB1*	c.171delG	p.N58TfsTer107	-No -No	Central nuclear Sx age 4	Parental (both parents carriers)	Pathogenic	-Female/10 years -Male/10 Years	Cohen et al. [20]
FAM13 -II:1	Ashkenazi	AD	-	Panel + MLPA	*GJA8*	Heterozygote duplication in the distal region of 1q21.11 that includes the genes PRKAB2, FMO5, BCL9, ACP6, GJA5, GJA8		-No	-Dense Cortical Surgery age 2–3 months	De novo	Likely pathogenic	-Female/1year	Brunetti-Pierri et al. [21]
FAM14 -II:1	Muslim	X-linked Dominant	-	Panel	*BCOR*	c.3985_3986del (het)	p. C1329Qfs*45	-Yes	-PFV, microphthalmia, ear malformation, VSD/ASD Sx age 3 months	Paternal (unaffected)	Pathogenic	-Female/6 months	Ng et al. [22]
FAM15 -II:1	Ashkenazi	Unknown	-	Panel	No gene			N/A	N/A *	No gene identified		Female/ 29 years	
FAM16 -I:1 -II:1	Ashkenazi-Yemen	AD	+	Exome	*GJA3*	c.199G > T,	p.D67Y	-Yes -Yes	- N/A -dense nuclear and capsular *	Paternal (affected)	Pathogenic	-Male-43 years -Male-	Boese et al. [23]
FAM17 -II:2	Morocco-Libya	AR	+	Panel	*CYP27A1*	c.845-1G > A (het)/ c.1016 C > T (het)	Splice site/ p.T339M	-No -No	--Post capsular-developmental. Surgery age 7 years CTX	Parental (both parents carriers)	Both pathogenic	-Male/6 years -Male/8 years	Leitersdorf et al. [24], Reshef et al [25]
FAM18 -I:1 -II:2	Jewish	AD	+	Sanger	*PAX6*	c.97G > C	p.A33P	-No -No	---Ant polar Mild corectopia *	Maternal (affected)	Pathogenic	-Female -Female -Male /35 years	Hanson et al. [26]
FAM19 -II:1	Ashkenazi	Unknown	-	Panel	No gene			N/A	Dense cataract-sx age 4 months. *	No gene identified		Female/29 years	
FAM20 -I:1 -II:2	Muslim	AD	(+) Mom and sister	?	*PAX6*	c.1310 A > T	p.Ter437L	-No -No	Anterior polar CAT Mild corectopia PANNUS FOVEAL HYPOPLASIA.	N/A	Pathogenic	-Female -Female -Male/5 years	Souzeau et al. [27]
FAM21 -I:2 -II:1	Ashkenazi	AD	+	Panel	*CRYBB2*	c.434G > C	p.R145P	-N/A -No	--Dense Nuclear cataract microphthalmia, sx age 2 months	Paternal (affected)	Likely pathogenic	-Male -Male -Female /3 months	Novel
FAM22 -I:1 -II:7	Ashkenazi	AD	+	Sanger	*PAX6*	c.77G > C	p.R26P	-No -No	-Ant polar + post sub-capsular. Surgery: age 8 years	Paternal (affected)	Pathogenic	-Male -2 male + 5 female/8 years	Redeker [28]

-* Genetic testing requested for family planning

-N/A: Not available

-No gene: no pathogenic or likely pathogenic variant identified

Genetic testing: Blood samples were collected for genetic testing from index patients. DNA was extracted using a DNA extraction kit (invitrogen. Molecular tests performed included NGS (i.e., whole exome sequencing and gene panel), Sanger sequencing, chromosomal microarray (CMA) and Multiplex Ligation-dependent Probe Amplification (MLPA), depending on patients’ preference and/or entity covering for genetic tests costs (HMO or private insurance). Blood samples from related family members were collected following identification of a causing pathogenic/likely pathogenic variant in the index patient. No instances of incomplete penetrance were identified.

A list containing the genes included in the panel-NGS based test associated with congenital cataract is provided as Supplementary material. Sanger sequencing was used for verification of variants as well as for segregation analysis.

Causative gene variants were identified in 18/22 families, including three novel and 15 known variants. Table 1 shows the gene variants spectrum found among our patients. Inheritance pattern was autosomal dominant (AD) in 13 out of 18 families, followed by X-linked dominant pattern in 3 of 18 families and autosomal recessive (AR) in two families.

Identified gene variants were mainly in crystalline genes (6/18 families; *CRYBA1, CRYBB1, CRYBB2, and CRYGD genes*) and in transcription factor genes (*PAX6 and BCOR*) in another 7/18 families (Table 2). Notably, 11 families performed genetic testing seeking pregnancy planning, of whom 7 had a causing gene identified and proceeded to pre-natal planning (Table 1).

**Table 2:** Gene’s spectrum causing bilateral pediatric cataract

Gene	Number of families identified	Ethnicity		Gene function	Syndrome
		MUSLIM ARABS	JEWISH		
CRYBA1	2		2	Crystalline	
CRYBB1	1	1			
CRYBB2	2		2		
CRYGD	1		1		
PAX6	4	1	3	Transcription factor gene	
BCOR	3	1	2		*
GJA3	1		1	Connexin	
GJA8	1		1		
MIP	2	1	1	Cytoskeleton protein	
CYP27A1	1		1		*

The phenotypic characteristics of cataracts varied among studied patients. Mild anterior polar opacities, with minimal visual axis obstruction was seen in four families with *PAX6* variants with only one patient requiring surgery at the age of 8 years (FAM22).

Progressive developmental nuclear cataract with two different identified genes, *CRYBB1* (FAM12) and *CYP27A1* (FAM17) caused moderate reduction in vision in two families (2 children each) needing surgery between four and seven years of age.

Dense lens opacities occluding visual axis that required early surgery (six months or earlier) were seen in seven families, two of which (FAM3 and FAM14) had a bilateral persistent fetal vasculature (PFV) cataract with an identified *BCOR* variant. Two families had a crystalline lens variant (FAM11 and FAM21) and another two families had a variant in a connexin gene (FAM13 and FAM16) (Table 1).

Cataract was believed to be isolated in all four families with *PAX6* variants as well as in one family with *BCOR* and another with *CYP27A1* variants; the accompanying ocular or systemic components were connected to the cataract only following genetic testing in these families.

Glaucoma was diagnosed in five of the 22 families; three of those had a *BCOR* gene variant. (Table 1).

## Discussion

Genetic counseling and testing became during the last years a significant clinical service for families affected by an inherited eye condition. Molecular testing are not considered anymore for “research purposes” but more an essential part of the ophthalmological service. Patients get to understand the origin, transmission paths and possibilities of prevention.

Our study highlights the importance of accessible genetic testing for families with BPC. Variant rate detection varied in our two different ethnic groups, reaching 100% (4/4) in Muslim families while among Jewish families it was 77.7% (14/18).

Variants in crystalline genes were prevalent among isolated BPC, representing a significant percentage of the families in our cohort (6 out of 18) consistent with the existing literature [9, 27, 29]. Moreover, transcription factor genes (specifically *PAX6 and BCOR*) were detected in a substantial proportion (7 out of 18) among our studied families, surpassing the prevalence reported in prior studies [30]. 

Dense nuclear cataracts were identified in association with crystalline genes (*CRYBA1, CRYBB2*) as previously described [30] but also with *connexin* and *BCOR* genes in our population.

The co-occurrence of BPC and glaucoma was observed in 5 out of 22 families: 3 families exhibited a *BCOR* gene variant, while one family presented the recently reported *GJA3* gene variant (c.199G > T), that has been associated with the co-occurrence of congenital cataract and glaucoma [21]. 

Patients in whom a variant was identified in the *PAX6* gene were referred for genetic testing due to anterior polar cataract with or without nystagmus, and no other apparent ocular pathology. Upon thorough ocular examination at our center, mild corectopia and foveal hypoplasia were observed. One patient (FAM22) needed cataract extraction at the age of 8 years due to progressive posterior capsular opacification in addition to anterior polar cataract. This is in accordance with the literature describing a highly variable phenotype in PAX6 variants[] [31–33].

Syndromic involvement was identified in 4/22 families (18%), of which 3/4 had a *BCOR* variant causing OFCD syndrome. Interestingly, 2/3 families (3 females) with a different *BCOR* gene variant identified, had PFV type of cataract, needing early surgery. In one of these families (FAM3) a girl was born with a bilateral cataract with PFV and no obvious systemic pathology noticed during the first years of age. Her mother had a unilateral cataract that was believed not to be inherited. Upon molecular testing a variant was identified in the *BCOR* gene in the girl and later, mosaicism was found in the mother. To the best of our knowledge, the association between *BCOR* gene and PFV has not been previously reported in the literature.

In the fourth family with a syndromic involvement two variants in the *CYP27A1* gene were identified reaching the diagnosis of cerebrotendinous-xanthomatosis (CTX). This family was under follow up due to bilateral developmental cataract in 2/6 of their children, with a background of delayed development in these children and another 2 siblings. Referral for genetic testing was done several times by treating ophthalmologists but parents did not comply. They opted to perform molecular testing when their children were eight and six years of age, finally reaching a molecular diagnosis. Of note, the variant *CYP27A1*: c.845-1G > A identified in this family, is a founder mutation in Moroccan Jews [22, 31]. 

The late molecular diagnosis at 6-to-8 years of age, such as in FAM17, can be preoccupying since many bilateral cataracts in infancy might be associated with a systemic condition or syndrome that can affect the child’s development or life span such as CTX, which has an existing treatment (chenodeoxycholic acid) that can prevent occurrence or progression of clinical manifestations of the disease if administered early enough [34, 35]. This fact enhances the importance of genetic testing in children with bilateral cataracts.

In addition, knowing the molecular etiology of a hereditary ocular disease in the family may also allow pre-marital counseling in certain populations as well as prenatal diagnosis and preimplantation genetic testing (PGT) in couples willing to do it [36]. 

We report three novel variants (two in *CRYBB2* gene and one in *MIP* gene) that were all categorized as “likely pathogenic” by the responsible molecular laboratory. In two among these families, segregation showed an affected parent.

Our study has limitations such as its retrospective nature. Identified gene variants did not undergo functional analysis, and in FAM9 (with a novel variant), parents did not complete segregation tests as requested.

In conclusion, combined molecular techniques showed a high detection rate of causative gene variants in our population. The confirmation of pathogenic variants can help in family counseling regarding recurrence risks and family planning, and, in some cases may aid in the diagnosis of a non-suspected syndromic type of pediatric cataract.

Our results suggest that BCOR gene variants might be associated with a PFV cataract. Further research is warranted to explore and establish any potential link between the *BCOR* gene and PFV cataract.

## Electronic supplementary material

Below is the link to the electronic supplementary material.

Supplementary Material 1
