# Supplementary material for: Genetics of bilateral pediatric cataract in the Israeli and Palestinian populations
Source: Graefes Arch Clin Exp Ophthalmol. 2024 Jun 14;262(10):3385–91. doi: 10.1007/s00417-024-06546-2 (PMC11458781; doi:10.1007/s00417-024-06546-2)
Supplement: Supplementary file 1 — Supplementary Material 1 [file 417_2024_6546_MOESM1_ESM.docx]

Genes included in panel NSG-based test for pediatric cataract

| ABCA3 | CYP51A1 | MYH9 | TDRD7 |
| --- | --- | --- | --- |
| ABCB6 | EPG5 | NDP | TFAP2A |
| ABHD12 | EPHA2 | NF2 | TMEM70 |
| ADAMTS18 | ERCC2 | NHS | UNC45B |
| ADAMTSL4 | ERCC5 | OCRL | VIM |
| AGK | ERCC6 | OPA3 | VSX2 |
| ALDH18A1 | ERCC8 | P3H2 | WDR87 |
| BCOR | EYA1 | PAX6 | WFS1 |
| BEST1 | FAM126A | PEX10 | WRN |
| BFSP1 | FOXC1 | PEX11B | XYLT2 |
| BFSP2 | FOXE3 | PEX16 | Collapse genes |
| CHMP4B | FTL | PEX2 |  |
| CLN3 | FYCO1 | PEX7 |  |
| COL11A1 | FZD4 | PITX2 |  |
| COL18A1 | GALK1 | PITX3 |  |
| COL2A1 | GALT | PXDN |  |
| COL4A1 | GCNT2 | RAB18 |  |
| COL4A2 | GFER | RAB3GAP1 |  |
| CRYAA | GJA1 | RAB3GAP2 |  |
| CRYAB | GJA3 | RDH11 |  |
| CRYBA1 | GJA8 | RECQL4 |  |
| CRYBA4 | HMX1 | RGS6 |  |
| CRYBB1 | HSF4 | RNLS |  |
| CRYBB2 | JAM3 | RRAGA |  |
| CRYBB3 | LEMD2 | SC5D |  |
| CRYGB | LIM2 | SIL1 |  |
| CRYGC | LONP1 | SIPA1L3 |  |
| CRYGD | LSS | SIX6 |  |
| CRYGS | MAF | SLC16A12 |  |
| CTDP1 | MIP | SLC33A1 |  |
| CYP27A1 | MIR184 | TBC1D20 |  |
|  |  |  |  |
